# The effects of a partitioned *var *gene repertoire of *Plasmodium falciparum *on antigenic diversity and the acquisition of clinical immunity

**DOI:** 10.1186/1475-2875-7-18

**Published:** 2008-01-23

**Authors:** Mario Recker, Nimalan Arinaminpathy, Caroline O Buckee

**Affiliations:** 1 Department of Zoology, South Parks Road, OX1 3PS, Oxford, UK; 2 Kenya Medical Research Institute, Centre for Geographic Medicine Research, Coast, Kilifi, Kenya

## Abstract

**Background:**

The human malaria parasite *Plasmodium falciparum *exploits antigenic diversity and within-host antigenic variation to evade the host's immune system. Of particular importance are the highly polymorphic *var *genes that encode the family of cell surface antigens PfEMP1 (*Plasmodium falciparum *Erythrocyte Membrane Protein 1). It has recently been shown that in spite of their extreme diversity, however, these genes fall into distinct groups according to chromosomal location or sequence similarity, and that recombination may be confined within these groups.

**Methods:**

This study presents a mathematical analysis of how recombination hierarchies affect diversity, and, by using simple stochastic simulations, investigates how intra- and inter-genic diversity influence the rate at which individuals acquire clinical immunity.

**Results:**

The analysis demonstrates that the partitioning of the *var *gene repertoire has a limiting effect on the total diversity attainable through recombination and that the limiting effect is strongly influenced by the respective sizes of each of the partitions. Furthermore, by associating expression of one of the groups with severe malaria it is demonstrated how a small number of infections can be sufficient to protect against disease despite a seemingly limitless number of possible non-identical repertoires.

**Conclusion:**

Recombination hierarchies within the *var *gene repertoire of *P. falciparum *have a severe effect on strain diversity and the process of acquiring immunity against clinical malaria. Future studies will show how the existence of these recombining groups can offer an evolutionary advantage in spite of their restriction on diversity.

## Background

Although the acquisition of immunity to malaria is poorly understood, it appears to rely on repeated exposure to different antigenic variants of the malaria parasite *Plasmodium falciparum *(for a review see [1,2]). Adults living in an endemic region will, therefore, have been exposed to, and generated antibodies to, most of the variants circulating in the parasite population [3-6] (although this is also dependent on transmission intensity, see for example [7,8]). In spite of this gradual accumulation of immunity with age and exposure, young children seem to be protected against the most severe forms of the disease after only a few episodes [9].

One of the main targets of protective immune responses against *P. falciparum *are the highly polymorphic variant surface antigens (VSA) [3,10-13], such as the *Plasmodium falciparum *Erythrocyte Surface Proteins, PfEMP1. These proteins are important virulence factors as they mediate cytoadherence to a variety of host cells receptors and cause sequestration of infected erythrocytes in vital organs such as the brain or placenta, which is a key element in the pathology of malaria [14-17]. PfEMP1 is encoded by a family of about 50–60 highly variable *var *genes per genome and is mutually exclusively expressed on the surface of infected red blood cells in a process known as clonal antigenic variation [18-21]. On a population level, the enormous sequence diversity of *var *genes facilitates the sequential reinfection of hosts, with the antigenic profile of newly infecting parasites appearing to correspond to a 'hole' in the antibody repertoire of the host [22-24]. Thus, immune responses elicited by one infection may not provide protection against a pathogen with a different set of *var *genes or a similar pathogen expressing a different subset of it *var *gene repertoire [11].

The extensive sequence diversity of PfEMP1 observed in the field is predominantly a consequence of high allelic and ectopic recombination rates [25-28]. However, the sequencing of the 3D7 malaria lab strain revealed genetic structuring in which *var *genes can be grouped according to their 5' upstream sequence (Ups) and chromosomal location [29], dividing them into into three major groups (A, B and C) and two intermediate groups (B/A and B/C) [30-32]. Bull *et al*. [22] proposed a different grouping based on features of short sequence tags within the Duffy-binding-like (DBL) *a *domain which can be sampled from most *var *genes using universal primers [28]. Even though this grouping is based on only a small portion of the gene it corresponds well with the ones based on whole genome sequences and it has become apparent that recombination is, at least to some extent, confined within these groups. Through the comparison of different *P. falciparum *isolates from diverse geographical regions, for example, Kraemer *et al*. [32] illustrated that these groups may be evolving independently of each other. Furthermore, it appears that these groups may have clinical significance, possibly due to functional differences. Several studies have found that severe disease in young children associates with the expression of *var *group A and B/A [33-35]. Rosetting is another well-known virulence factor and recent studies have identified associations between the rosetting phenotypes and the expression of particular groups of *var *genes [22,36,37]. These studies suggest that a relatively confined group of genes are responsible for most of the clinical infections cause by *P. falciparum*; this may also explain why immunity to clinical malaria is acquired early on in life (depending on transmission intensity) and after only a few episodes [9].

Understanding the implications for restricted recombination between groups of *var *genes is key for a proper assessment of PfEMP1 as a vaccine candidate [2,38]. For example, if there exists a small genetically isolated partition that can be associated with severe disease, this subset would be a good vaccine candidate in much the same way that *var*2*CSA*, whose expression has been associated with the phenomenon of pregnancy associated malaria [39,40], is being used as the basis for developing a pregnancy associated malaria vaccine (see [41] for a review of the progress). However, not much is known about the direct consequences of a recombination hierarchy on the acquisition of immunity and the diversity of the global *var *gene population. This question is being explored here by first demonstrating that any partitioning of the antigenic repertoire into genetically isolated groups has a limiting effect on the total diversity that can be created through recombination, and then by analysing how diversity affects an individual's development of immunity to clinical malarial disease.

## Methods and Results

**Figure 1:**
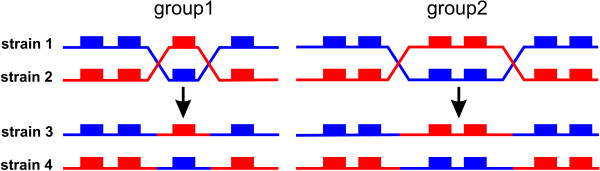
**Homologous recombination between two strains**. Recombination between two strains (strain 1, blue, and strain 2, red), shown as two cross-over events, is considered to be confined within recombining groups (here group1 and group2). Each box represents a particular gene. In this process, two strains emerge (strain 3 and strain 4) that differ in their antigenic repertoire from their parent strains.

In the following, an antigenic type or strain of *P. falciparum *is defined by its full repertoire of *var *genes. Two strains will then be said to have an overlapping repertoire if they have one or more *var *genes in common. In accordance with a recent model of antigenic variation in malaria, it is assumed that each PfEMP1 contains a unique major, immunogenic epitope [42]. The epitope itself might be encoded by a single combination of polymorphic building blocks within the gene such that each new combination, as a result of ectopic recombination between two genes, generates a novel epitope. For reason of simplicity, it is assumed that the population is at equilibrium with regards to these epitopes and every possible combination is currently in circulation within the parasite population. Although this assumption leads to an overestimation of diversity, it allows us to carry out the following analysis in disregard of ectopic recombination and generation of new variants. Therefore, recombination is considered only between two *P. falciparum *strains when it results in the generation of novel strains that differ in their antigenic repertoire from their 'parent' strains; this concept is illustrated in Figure 1.

Diversity is considered at two separate levels: intra-genic and inter-genic. The degree of intra-genic diversity, defined as *n*, refers to the number of unique epitopes at the genome level, whereas inter-genic diversity, defined as *N*, refers to the number of epitopes at the population level. Because each *var *gene is assumed to contain a single unique epitope, 'gene' and 'epitope' are interchangeable in this context.

### Diversity reduction through segmentation

Each *P. falciparum *genome contains around 60 *var *genes, i.e. 60 unique epitopes. The extent of diversity at the population level, however, is much higher. As a result, allelic recombination between distinct *P. falciparum *strains can lead to an enormous number of different combinations of genes. As a numerical example, with 800 distinct epitopes within a local setting the number of possible non-identical repertoires that can be created through recombination without constraints, referred to as *D*_*T*_, is given as

$$D_{T}=\left(\begin{array}{c} 800\\ 60 \end{array}\right)=\frac{800!}{60!(800-60)!}\approx 1.9\cdot 10^{91}.$$

As a consequence of dividing the antigenic repertoire into recombining groups, epitopes are only available to the groups they are associated with. If the repertoire is divided into two partitions or recombining groups, such that *n*_1 _+ *n*_2 _= *n *and *N*_1 _+ *N*_2 _= *N*, the number of possible strains is given by

$$D_{P}=\left(\begin{array}{c} N_{1}\\ n_{1} \end{array}\right)\cdot \left(\begin{array}{c} N_{2}\\ n_{2} \end{array}\right).$$

However, the pool size of possible strains that can be created from genomes in which recombination is partitioned is always smaller than the pool size that can be created from the same number of epitopes in a non-partitioned system, that is,

$$\left(\begin{array}{c} N_{1}\\ n_{1} \end{array}\right)\cdot \left(\begin{array}{c} N_{2}\\ n_{2} \end{array}\right)<\left(\begin{array}{c} N_{1}+N_{2}\\ n_{1}+n_{2} \end{array}\right).$$

Therefore, recombination hierarchies have a limiting effect on the level of strain diversity. More importantly, the finer the partitioning the greater is the limiting effect. As an example, assuming that the repertoire is equally divided into five recombining groups such that each group has the same number of epitopes, both within the genome and within the population, i.e. *n*_*i *_= *n/*5 = 12 and *N*_*i *_= *N/*5 = 160. The reduction in diversity with this grouping can be calculated by the ratio *D*_*P*_/*D*_*T *_(i.e. the number of combinations with partitioning divided by the number of combinations without partitioning):

$$\frac{D_{P}}{D_{T}}={\left(\begin{array}{c} n\\ N \end{array}\right)}^{-1}\prod_{i=1..5}\left(\begin{array}{c} n_{i}\\ N_{i} \end{array}\right)<5\cdot 10^{-4}.$$

This means that the potential pool size would be reduced by nearly four orders of magnitude by such a partitioning. Note that the existence of recombining blocks also restricts the number of possible epitopes that can be created through ectopic recombination (because the availability of 'building blocks' is limited); this puts a further limitation on the number of possible strains. Therefore, the diversity reduction ratio serves only as a lower bound for the constraining effect of recombination hierarchies.

### Intra- and inter-genic diversity

Allocating genes or epitopes to distinct recombining groups limits the total number of strains but the group size distribution itself determines the extent to which strain diversity is constrained. For example, if a total pool of 800 epitopes, with 60 epitopes per genome, is divided into two equally sized recombining groups, the reduction in diversity is nearly twice as much as if one group was 10 times bigger than the other group. (Naturally, in the extreme case effectively equals an unconstrained system which has the highest level of diversity.) Therefore, the number of strains that can be created in a partitioned system is greater if the partitions are unequal in size; this holds true for any number of partitions.

**Figure 2:**
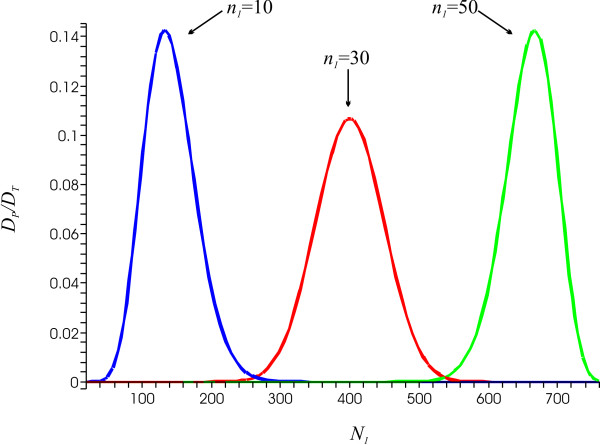
**Diversity reduction**. The diversity reduction ratio *D*_*P*_/*D*_*T *_crucially depends on the relationship between the respective intra to inter-genic diversity ratios *n*_*i*_/*N*_*i*_, and is minimised exactly when *n*_1_/*N*_1 _= *n*_2_/*N*_2_. A small *D*_*P*_/*D*_*T *_ratio indicates a greater reduction in diversity. *n *= *const *= 60 and *N *= *const *= 800 (*n*_1 _+ *n*_2 _= *n *and *N*_1 _+ *N*_2 _= *N*).

What about the relationship between the intra and inter-genic group sizes? Bull *et al*. [22] reported similar frequency distributions of groups of *var *sequence tags within the parasite genome and within the whole population despite the fact that the groups themselves vary considerably in size. That is, the relative ratios of intra to inter-genic group sizes, *n*_*i*_/*N*_*i*_, are equal across the groups. Assuming a population pool of *N *epitopes, with each parasitic genome containing *n *epitopes grouped into two major groups, *n*_1 _and *n*_2_, one can calculate the diversity reduction *D*_*P*_/*D*_*T *_for varying intra to inter-genic group size ratios, *n*_1_/*N*_1 _and *n*_2_/*N*_2_. Figure 2 shows how the diversity reduction ratio *D*_*P*_/*D*_*T *_changes with inter-genic group size *N*_1 _for three different values of *n*_1 _for a system *N *= 800 and *n *= 60 (*n*_2 _and *N*_2 _are determined by *n*_2 _= *n - n*_1 _and *N*_2 _= *N - N*_1_). In all three cases, *D*_*P*_/*D*_*T *_assumes a maximum exactly when *n*_1_/*N*_1 _= *n*_2_/*N*_2_. For example, in the case where *n*_1 _= 10 and *n*_2 _= 50, the smallest reduction in strain diversity is found when *N*_1 _= 133 and *N*_2 _= *N - N*_1 _= 800 - 133 = 667, i.e. when *n*_1_/*N*_1 _= *n*_2_/*N*_2 _= 1/5. This clearly demonstrates that the number of possible strains the parasite can create through recombination is least restricted (greatest value of *D*_*P*_/*D*_*T*_) when the frequency distributions of the different groups are equal within parasite genomes and across the whole population. Due to symmetry, the same results can be obtained for any number of partitions.

### Overlapping repertoires

Immunity against severe malaria develops rapidly after relatively few clinical episodes, and recent studies have shown that severe disease may correlate with the expression of particular groups of *var *genes. Here it is assumed that each genome contains between six and 10 genes of this "severe disease" group and it is further assumed that each gene will be expressed during the course of infection. In the following, *n*_*SD *_and *N*_*SD *_define the number of severe disease (SD) associated genes within the genome and within the population, respectively. With an arbitrary intra to inter-genic diversity ratio *n*_*SD*_/*N*_*SD *_= 1/20, a pool of 120–200 SD-epitopes can be found within the population, and the boundaries for the total number of unique SD repertoires are given as

$$\begin{array}{ccc} D_{lower}=\left(\begin{array}{c} 120\\ 6 \end{array}\right)=3.65\cdot 10^{9} & \text{and} & D_{upper}=\left(\begin{array}{c} 200\\ 10 \end{array}\right)=2.45\cdot 10^{16} \end{array}.$$

¿From now on, only epitopes associated with severe disease are being considered which allows the omission of the subscripts, i.e. *n*_*SD *_= *n *and *N*_*SD *_= *N*. Although the number of possible combinations of epitopes is huge, a large proportion of these have one or more epitopes in common. The proportion of combinations, i.e. strains, that share *i *epitopes can be expressed as

$$s_{i}=\left(\begin{array}{c} n\\ i \end{array}\right)\left(\begin{array}{c} N-n\\ n-i \end{array}\right){\left(\begin{array}{c} N\\ n \end{array}\right)}^{-1}$$

and find the proportion of strains that overlap in their repertoire of severe disease associated genes by at least one as ∑_*i *_*s*_*i*_, which can also be expressed as

$$p_{\text{overlap}}=1-\left(\begin{array}{c} N-n\\ n \end{array}\right){\left(\begin{array}{c} N\\ n \end{array}\right)}^{-1},$$

where the last term denotes the proportion of types with no overlap. For the lower and upper bound systems, (*N, n*) = (120, 6) and (200, 10), the proportion of overlapping types is 27% and 41%, respectively. Therefore, under the assumption of equal intra to inter-genic diversity ratios, larger groups tend to produce more overlapping combinations as the probability of two types having epitopes in common increases with the size of the genomic repertoire.

The assumption that every gene will be expressed during infection is of course artificial, but serves to illustrate the counterintuitive concept that overlap between strains actually increases with diversity when the intra to inter-genic diversity ratio is approximately equal. This will be shown to have consequences for the rate at which individuals acquire immunity through a series of infections.

### Acquired immunity against severe disease

For an individual host suffering a series of infections, *e*_*t *_defines the number of SD epitopes he/she has been exposed to after the *t*^*th *^infection. Recall that each type contains *n *epitopes and so the probability of getting infected with *m *novel epitopes at the next infection, is equal to the proportion of strains that contain exactly *n - m *epitopes the host has previously been exposed to (note, it is assumed that all *n *genes will be expressed during the infection). That is,

$$\begin{array}{cc} P(e_{t+1}-e_{t}=m|e_{t})=\left(\begin{array}{c} e_{t}\\ n-m \end{array}\right)\left(\begin{array}{c} N-e_{t}\\ m \end{array}\right){\left(\begin{array}{c} N\\ n \end{array}\right)}^{-1}, & 0\leq m\leq n. \end{array}$$

**Figure 3:**
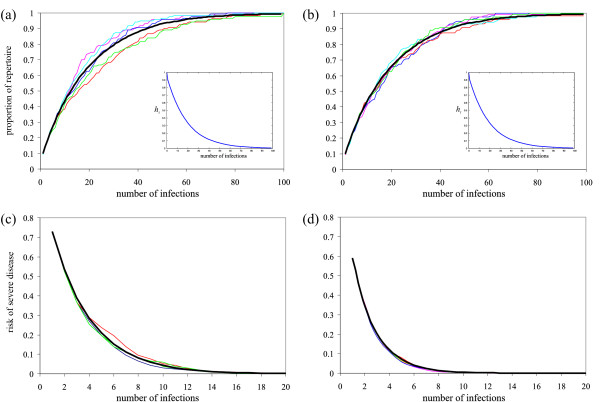
**Simulation of infection histories**. Stochastic simulation of five individual infection histories showing (A) the acquisition of immunity in terms of exposed upsA associated epitopes and (B) the drop in the risk of severe disease with the number of infections; the black lines are the averages of 100 simulations. Left panel: (*N, n*) = (120, 6), right panel: (*N, n*) = (200, 10). The insets show how the gap in the immune repertoire, *h*_*t*_, decreases with consecutive infections.

Moreover, *P*(*e*_1 _= *n*) = 1, as the first infection necessarily results in *n *novel epitopes. Figure 3 illustrates the result of a stochastic simulation of individual infection histories where the outcome of each infection is determined by (8). Figure 3(a) and 3(b) show the build-up of immunity in terms of exposed epitopes with infection history for the two systems (*N, n*) = (120, 6) and (200, 10). From these plots one can notice two phenomena: first, defining *h*_*t *_: = 1 - *e*_*t*_/*N *as the proportion of epitopes an individual has not yet experienced after the *t*^*th *^infection, i.e. the gap in its immune repertoire, then *h*_*t *_decays exponentially with *t *(shown as insets in Figure 3(a) and 3(b)). Second, the rate at which *h*_*t *_decays is equal for both systems (*N, n*) = (120, 6) and (200, 10). Therefore, the rate at which individuals get exposed to novel epitopes is mostly dependent on the ratio between the number of epitopes one experiences at each infection and the total number of epitopes within the population, *n/N*.

Here, it is assumed that the risk of developing severe disease corresponds to the probability of getting infected with an strain that does not contain any SD associated epitopes the host has previously been exposed to. Although a crude assumption, it is easy to see how the results obtained here can be applied to cases where minimal overlap can also cause severe disease or even to the case when not all genes are being expressed during infection. In terms of an individual's infection history the risk of severe disease is then given as the probability of experiencing *n *novel epitopes at the (*t *+ 1)^*th *^infection. That is,

$$P_{SD}=P(e_{t+1}-e_{t}=n|e_{t})=\left(\begin{array}{c} N-e_{t}\\ n \end{array}\right){\left(\begin{array}{c} N\\ n \end{array}\right)}^{-1}.$$

By simulating individual infection histories it can be shown how the risk of severe disease decreases with each infection *t*; Figure 3(c) and 3(d) show the result of the stochastic simulations. In line with the gap in the host's immune repertoire, *h*_*t*_, the risk of severe disease *P*_*SD *_decays exponentially with infection history, and it is worth mentioning that even after a limited number of infections the risk of getting infected by an entirely novel strain is minimal. For example, with 200 SD associated genes in the community and 10 per genome, the risk of severe disease is one in 1,000 after 13 infections only. However, it takes more than 22 infections if there are 120 SD genes in the community and six per genome, despite the fact that the total number of possible combinations in this case is less by over six orders of magnitude.

**Figure 4:**
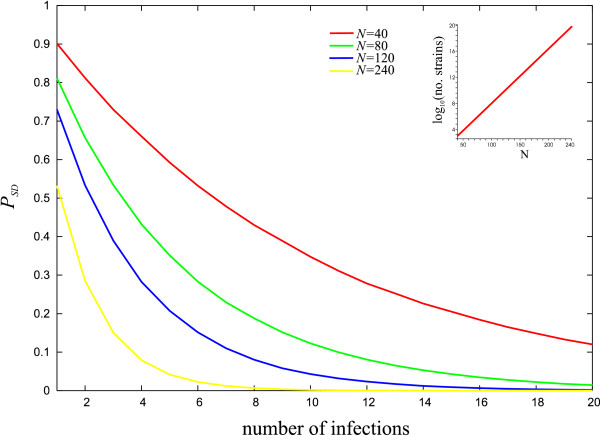
**Risk of severe disease**. Risk of severe disease at different levels of diversity *N*, with *n/N *= *const *= 0.05. As the level of inter-genic diversity increases the incremental risk of contracting a discordant strain decreases significantly. Each line represents the average of 100 simulations. The inset shows how the total number of strains (log scale) that can be generated through recombination increases with *N*.

In contrast to the acquisition of an immune repertoire (through exposure to novel epitopes), the change in risk of severe disease with infection history is strongly and separately dependent both on the intra and inter-genic diversity levels. Figure 4 shows how *P*_*SD *_depends on the genetic diversity of these epitopes in the population, *N*, keeping the intra to inter-genic diversity ratio, *n/N*, constant. A surprising effect of maintaining a constant *n/N *ratio is that enhanced diversity leads to a sharper drop in the risk of severe disease with every infection. For example, with 12 such genes per genome and 240 within the population, 11 infections can be sufficient to bring the risk of contracting a discordant type down to less than one in 1,000. On the other hand, with only six SD associated genes per genome and a total of 120 in the population it takes twice as many infections to gain the same level of protection.

This phenomenon occurs because larger groups tend to produce more overlapping combinations (under the assumption that the ratio between the number of epitopes per genome and the total number of epitopes in the population is constant). Furthermore, hosts experience a higher number of epitopes through each infection. As a result, after relatively few infections, the overall number of exposed epitopes is bigger than for smaller groups (again, under the assumption that all genes are being expressed). Because of the extensive overlap of strains in the population, the risk of getting infected by a novel combination decays more rapidly. Note, these results rest on the assumption that all epitopes are expressed during infection, although there is little data on *var *gene expression patterns *in vivo*. From Figure 4 it is clear, however, that the rate at which clinical protection is acquired strongly correlates with the number of genes that the host experiences per infection. If for example only a subset of genes is being expressed during infection or reaches a sufficient level to trigger an immune response, this would have a negative effect on the acquisition of immunity.

### Recombination and immune selection

**Figure 5:**
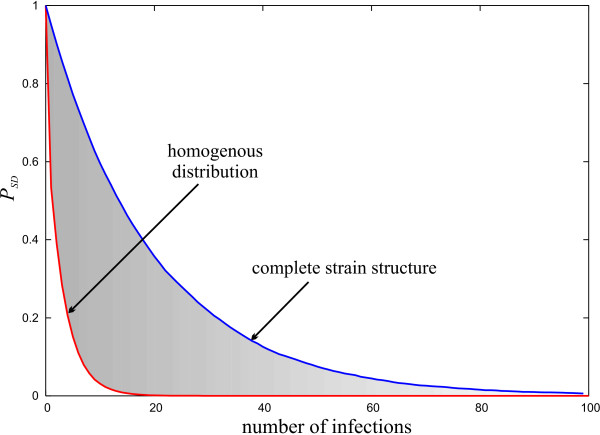
**Strain structured vs. homogenous population**. Comparison between the decline in risk of severe disease in a homogenous distribution of all possible types and a strain structured population. Both hypothetical extremes, homogenous distribution and complete strain structure, define the boundaries of a region (grey) within which the risk will realistically decay with exposure. *N *= 120, *n *= 6, *η *= 20.

So far it has been assumed that all possible combinations of *var *genes occur within the parasite population at equal prevalence. Another theoretical extreme can be found within a totally structured population. Mathematical models have shown that strong immune selection can cause antigenically diverse pathogen populations to segregate into sets of strains that are marked by discordant antigenic repertoires [43,44]. Within this framework, assuming strong selection pressure and an entirely discordant pathogen population, an estimate for the lower bound for the total diversity of strains is *η *: = *N/n*, i.e. the total number of unique *var *epitopes is divided into discrete, non-overlapping types. This hypothetical extreme of a completely strain structured population means that each infection is with a strain that is either novel or overlaps entirely with the ones the host has previously been exposed to. The probability of severe disease is then simply given as

$$P_{SD}=\frac{\eta -e}{\eta },$$

where *e *is the number of strains the host has experienced. However, a pathogen population that is structured into discordant types may actually lower the rate at which individuals build up protection against severe disease. Figure 5 shows how the risk of disease decreases with infection history under the assumption of total strain structure compared to that of a homogenous setting where all possible combinations are equally prevalent in the population. These two extremes define the boundaries of a region where the actual drop in the risk of severe disease can be expected. This intermediate region is very likely to be influence by the local settings, especially the rate and mode of transmission.

## Discussion and Conclusion

Despite different approaches to classifying the *var *genes of *P. falciparum*, it appears that recombination between genes and gene segments is, at least to some extent, confined within a number of groups. It has been speculated that this partitioning has evolved to accommodate various sets of genes with distinct antigenic and adhesive characteristics within each parasite genome [31,32]. Here it has been shown that confining recombination to specific groups also has a limiting effect on the overall level of diversity that can be attained by the parasite.

Within the model framework, an equal ratio of intra- to inter-genic diversity allows the parasite to generate a maximum level of strain diversity within the constraints of such a recombination hierarchy. In fact, the limiting effect of repertoire partitioning is strongly dependent on the number of partitions, their respective sizes and the distribution of epitopes among them. Interestingly, the lowest pathogen population diversity will be attained if the partitions are exactly equal in size, whereas pathogen populations with asymmetric group sizes are able to maintain a higher level of diversity. This prediction is consistent with studies showing wide variations among group sizes, although the sizes themselves seem remarkably stable. That maximum diversity is obtained when intra- to inter-genic diversity ratios are equal is particularly interesting in light of findings that the groups defined by [22] do seem to show very similar frequency distribution among the group members within a single parasite and the population as a whole, despite the fact that the relationship between the genomic group size and the total pool of genes associated with the group is non-linear. That is, the ratio between the number of genes associated with a particular group within the genome and the number of those genes within the population is approximately the same for every group. One of the main caveats in the analysis is the restriction to homologous recombination alone, however. Ectopic recombination is known to be a major generator of *var *gene diversity. It can be expected that the rate at which novel and functional genes are being created and introduced into the population is slower than homologous recombination. Therefore, the analysis can be seen as being applied to a snapshot of the current diversity within the population. The assumption that all possible combination exist within the population, however, might compensate for disregarding the generation of novel variants. The exact level of diversity within a population is not known, and it is also not known if diversity is constant, fluctuating or growing, or how fast the turnover of *var *genes is. These are important considerations for future work.

The practical implication of categorizing *var *genes is to find associations between certain disease symptoms and the expression of particular groups of genes. Several studies have already found correlations between specific groups and clinical manifestations, such as severe childhood disease or rosetting, although more research is required to establish the exact nature of these associations. For the purpose of this analysis it was assumed that each genome contained a certain number of disease associated genes and that clinical protection is the result of having been exposed to a majority of circulating variants. More precisely, the risk of severe disease was assumed to correlate with the probability of getting infected by a strain with a completely discordant set of these genes. This was done for practical reasons only, although it is conceivable that any pre-existing immunity, in terms of specific antibodies, is likely to offer some degree of protection. Furthermore, the process of acquired immunity is multi-factorial and depends on more than the exposure to PfEMP1. Host factors and other parasitic proteins, such as the vaccine candidate merozoite surface protein (MSP), might also be crucial during this process ([45-47]). The diversity of these proteins is far more limited than PfEMP1, however, such that within the model presented here, a much steeper reduction in the probability of developing clinically disease with each infection can be expected. The analytical framework presented here can easily be extended to include different levels of diversity to account for more than one target of protective immune responses. However, if both targets are equally strongly associated with protective immunity, then the least diverse target would dominate the rate at which protection is acquired.

In terms of the actual number of genes an individual is being exposed to during infection it was found that a larger group size, i.e. a larger number of genes per genome and a proportionally larger number of genes across the population, resulted in a more significant decrease in the risk of severe disease with each infection. This occurred for two reasons: (i) larger groups tended to produce a higher proportion of strains that overlap in the antigenic repertoire; this lowered the per-infection risk of encountering a completely novel strain. (ii) larger groups led to hosts being exposed to higher number of variants per infection, which resulted in a further decrease in risk. The analysis presented here is based on the crucial assumption that every gene will be expressed during infection. This, however, is biologically questionable. As a result, the gradient shown for the decline in the risk of severe disease is probably steeper than if only a certain portion of the repertoire is being expressed. Nevertheless, the model serves to illustrate how the reduction in risk of severe disease can fall rapidly in spite of vast pathogen diversity. Variant expression itself appears to be influenced by the immune status of the host [22]. Although no specific order has been established at which the variants are being expressed during infection, there is some evidence that antibodies against particular groups of genes (groups A and B/A) are acquired earlier in life, which hints towards a predominance of this particular group [8]. This apparent predominance might also help to explain why protection against the most severe form of malaria is acquired early on in life.

The gradient in the decline in risk of severe disease is also influenced by the parasite population structure. Theoretical models have suggested that immune selection can segregate *P. falciparum *into discrete strains with minimal overlap in their antigenic repertoire [43,44]. This has been supported by field studies that also found little or no overlap among DBL-*α *sequences between different isolates [48,49]. These studies are not entirely conclusive as they relied on a relatively small number of isolates or came from regions of low-moderate transmission intensity. The latter is expected to have a major influence on population structure as allelic recombination is hugely favoured when transmission is intense. Nevertheless, as Figure 5 clearly shows, population structures can have a huge impact on the rate at which individuals acquire protective immunity. Both hypothetical extremes, a strain structured population and one where all possible combinations co-circulate, can be seen as setting an upper and lower level for the rate at which the risk of severe disease decreases with infection history. The realistic rate can then be expected to be somewhere between those boundaries, determined by the intensity of transmission, for example.

The evolutionary forces that determine the size of each *var *gene group are not clear but one can envisage a complex and multi-factorial interplay of trade-offs between disease, infection length and host adaptation (including both immune evasion and cytoadhesion). For example, high levels of parasitaemia correlate with transmissibility but also with the risk of host death. Increasing the number of disease-associated genes might, therefore, be detrimental for the pathogen's fitness. On the other hand, retaining a certain number of these genes may confer an evolutionary advantage over parasites that carry only genes associated with mild disease because of increased transmissibility. This is highly speculative but the fact that clinical symptoms so far have only been found to correlate with the expression of a relatively small set of genes within the repertoire seems to support these claims. Ongoing studies will help to clarify how selection pressure has shaped and partitioned the *var *gene repertoire of *P. falciparum *and how it impacts on the expression of different subsets of genes. This knowledge is key to understanding host-pathogen interaction which might aid the design of new and ongoing intervention strategies.

## Authors' contributions

MR performed the analysis, carried out the stochastic simulations and wrote the manuscript. NA actively contributed to the analysis and helped to write the manuscript. COB helped to write the manuscript.

## References

[B1] Hviid L (2005). Naturally acquired immunity to *Plasmodium falciparum *malaria in Africa. Acta Trop.

[B2] Chen Q (2007). The naturally acquired immunity in severe malaria and its implication for a PfEMP-1 based vaccine. Microbes Infect.

[B3] Marsh K, Howard RJ (1986). Antigens induced on erythrocytes by P. falciparum: expression of diverse and conserved determinants. Science.

[B4] Bull P, Lowe B, Kortok M, Marsh K (1999). Antibody recognition of *Plasmodium falciparum *erythrocyte surface antigens in Kenya: Evidence for rare and prevalent variants. Infect Immun.

[B5] Bull P, Kortok M, Kai O, Ndungu F, Ross A, Lowe B, Newbold C, Marsh K (2000). Plasmodium falciparum-infected erythrocytes: Agglutination by diverse Kenyan plasma is associated with severe disease and young host age. J Infect Dis.

[B6] Ofori M, Dodoo D, Staalsoe T, Kurtzhals J, Koram K, Theander T, Akanmori B, Hviid L (2002). Malaria-induced acquisition of antibodies to *Plasmodium falciparum *variant surface antigens. Infect Immun.

[B7] Chattopadhyay R, Sharma A, Srivastava V, Pati S, Sharma S, Das B, Chitnis C (2003). *Plasmodium falciparum *infection elicits bothvariant-specific and cross-reactive antibodies against variant surface antigens. Infect Immun.

[B8] Joergensen L, Vestergaard LS, T L, Magistrado P, Lusingu JP, Lemnge M, Theander T, Jensen ATR (2007). 3D7-Derived *Plasmodium falciparum *erythrocyte membrane protein 1 is a frequent target of naturally acquired antibodies recognizing protein domains in a particular pattern independent of malaria transmission intensity. J Immunol.

[B9] Gupta S, Anderson R (1999). Population structure of pathogens: The role of immune selection. Parasitol Today.

[B10] Leech JH, Barnwell JW, Miller LH, Howard RJ (1984). Identification of a strain-specific malarial antigen exposed on the surface of *Plasmodium falciparum*-infected erythrocytes. J Exp Med.

[B11] Bull P, Lowe B, Kortok M, Molyneux C, Newbold C, Marsh K (1998). Parasite antigens on the infected red cell surface are targets for naturally acquired immunity to malaria. Nat Med.

[B12] Giha H, Staalsoe T, Dodoo D, Roper C, Satti G, Arnot D, Hviid L, Theander T (2000). Antibodies to variable *Plasmodium falciparum*-infected erythrocyte surface antigens are associated with protection from novel malaria infections. Immunol Lett.

[B13] Dodoo D, Staalsoe T, Giha H, Kurtzhals J, Akanmori B, Koram K, Dunyo S, Nkrumah F, Hviid L, Theander T (2001). Antibodies to variant antigens on the surfaces of infected erythrocytes are associated with protection from malaria in Ghanaian children. Infect Immun.

[B14] Barnwell JW, Asch AS, Nachman RL, Yamaya M, Aikawa M, Ingravallo P (1989). A human 88-kD membrane glycoprotein (CD36) functions in vitro as a receptor for a cytoadherence ligand on *Plasmodium falciparum*-infected erythrocytes. J Clin Invest.

[B15] Berendt AR, Simmons DL, Tansey J, Newbold CI, Marsh K (1989). Intercellular adhesion molecule-1 is an endothelial cell adhesion receptor for *Plasmodium falciparum*. Nature.

[B16] Turner G, Morrison H, Jones M, Davis T, Looareesuwan S, Buley I, Gatter K, Newbold C, Pukritayakamee S, Nagachinta B (1994). An immunohistochemical study of the pathology of fatal malaria. Evidence for widespread endothelial activation and a potential role for intercellular adhesion molecule-1 in cerebral sequestration. Am J Pathol.

[B17] Rogerson SJ, Chaiyaroj SC, Ng K, Reeder JC, Brown GV (1995). Chondroitin sulfate A is a cell surface receptor for *Plasmodium falciparum*-infected erythrocytes. J Exp Med.

[B18] Roberts D, Craig A, Berendt A, Pinches R, Nash G, Marsh K, Newbold C (1992). Rapid switching to multiple antigenic and adhesive phenotypes in malaria. Nature.

[B19] Su X, Heatwole V, Wertheimer S, Guinet F, Herrfeldt J, Peterson D, Ravetch J, Wellems T (1995). The large diverse gene family var encodes proteins involved in cytoadherence and antigenic variation of *Plasmodium falciparum*-infected erythrocytes. Cell.

[B20] Scherf A, Hernandez-Rivas R, Buffet P, Bottius E, Benatar C, Pouvelle B, Gysin J, Lanzer M (1998). Antigenic variation in malaria: in situ switching, relaxed and mutually exclusive transscription of var genes during intra-erythrocytic development in *Plasmodium falciparum*. EMBO J.

[B21] Newbold C (1999). Antigenic Variation in *Plasmodium falciparum *: Mechanisms and consequences. Curr Opin Microbiol.

[B22] Bull PC, Berriman M, Kyes S, Quail MA, Hall N, Kortok MM, Marsh K, Newbold CI (2005). *Plasmodium falciparum *variant surface antigen expression patterns during malaria. PLoS Pathog.

[B23] Barry A, Leliwa-Sytek A, Tavul L, Imrie H, Migot-Nabias F, Brown S, McVean G, Day K (2007). Population genomics of the immune evasion (var) genes of *Plasmodium falciparum*. PLoS Pathog.

[B24] Volkman SK, Sabeti PC, DeCaprio D, Neafsey DE, Schaffner SF, Milner DAJ, Daily JP, Sarr O, Ndiaye D, Ndir O, Mboup S, Duraisingh MT, Lukens A, Derr A, Stange-Thomann N, Waggoner S, Onofrio R, Ziaugra L, Mauceli E, Gnerre S, Jaffe DB, Zainoun J, Wiegand RC, Birren BW, Hartl DL, Galagan JE, Lander ES, Wirth DF (2007). A genome-wide map of diversity in *Plasmodium falciparum*. Nat Genet.

[B25] Conway DJ, Roper C, Oduola AM, Arnot DE, Kremsner PG, Grobusch MP, Curtis CF, Greenwood BM (1999). High recombination rate in natural populations of *Plasmodium falciparum*. Proc Natl Acad Sci USA.

[B26] Ward C, Clottey G, Dorris M, Ji D, Arnot D (1999). Analysis of *Plasmodium falciparum *PfEMP-1 var genes suggests that recombination rearranges constrained sequences. Mol Biochem Parasitol.

[B27] Freitas-Junior LH, Bottius E, Pirrit LA, Deitsch KW, Scheidig C, Guinet F, Nehrbass U, Wellems TE, Scherf A (2000). Frequent ectopic recombination of virulence factor genes in telomeric chromosome clusters of *P. falciparum*. Nature.

[B28] Taylor H, Kyes S, Newbold C (2000). Var Gene Diversity in *Plasmodium falciparum *is generated by frequent recombination events. Mol Biochem Parasitol.

[B29] Gardner MJ, Hall N, Fung E, White O, Berriman M, Hyman RW, Carlton JM, Pain A, Nelson KE, Bowman S, Paulsen IT, James K, Eisen JA, Rutherford K, Salzberg SL, Craig A, Kyes S, Chan MS, Nene V, Shallom SJ, Suh B, Peterson J, Angiuoli S, Pertea M, Allen J, Selengut J, Haft D, Mather MW, Vaidya AB, Martin DMA, Fairlamb AH, Fraunholz MJ, Roos DS, Ralph SA, McFadden GI, Cummings LM, Subramanian GM, Mungall C, Venter JC, Carucci DJ, Hoffman SL, Newbold C, Davis RW, Fraser CM, Barrell B (2002). Genome sequence of the human malaria parasite *Plasmodium falciparum*. Nature.

[B30] Lavstsen T, Salanti A, Jensen ATR, Arnot DE, Theander TG (2003). Sub-grouping of *Plasmodium falciparum *3D7 var genes based on sequence analysis of coding and non-coding regions. Malar J.

[B31] Kraemer SM, Smith JD (2003). Evidence for the importance of genetic structuring to the structural and functional specialization of the *Plasmodium falciparum *var gene family. Mol Microbiol.

[B32] Kraemer SM, Kyes SA, Aggarwal G, Springer AL, Nelson SO, Christodoulou Z, Smith LM, Wang W, Levin E, Newbold CI, Myler PJ, Smith JD (2007). Patterns of gene recombination shape var gene repertoires in *Plasmodium falciparum *: comparisons of geographically diverse isolates. BMC Genomics.

[B33] Jensen ATR, Magistrado P, Sharp S, Joergensen L, Lavstsen T, Chiucchiuini A, Salanti A, Vestergaard LS, Lusingu JP, Hermsen R, Sauerwein R, Christensen J, Nielsen MA, Hviid L, Sutherland C, Staalsoe T, Theander TG (2004). *Plasmodium falciparum *associated with severe childhood malaria preferentially expresses PfEMP1 encoded by group A var genes. J Exp Med.

[B34] Kyriacou HM, Stone GN, Challis RJ, Raza A, Lyke KE, Thera MA, Kone AK, Doumbo OK, Plowe CV, Rowe JA (2006). Differential var gene transcription in *Plasmodium falciparum *isolates from patients with cerebral malaria compared to hyperparasitaemia. Mol Biochem Parasitol.

[B35] Rottmann M, Lavstsen T, Mugasa JP, Kaestli M, Jensen ATR, Muller D, Theander T, Beck HP (2006). Differential expression of var gene groups is associated with morbidity caused by *Plasmodium falciparum *infection in Tanzanian children. Infect Immun.

[B36] Kaestli M, Cockburn IA, Cortes A, Baea K, Rowe JA, Beck H (2006). Virulence of malaria is associated with differential expression of *Plasmodium falciparum *var gene subgroups in a case-control study. J Infect Dis.

[B37] Normark J, Nilsson D, Ribacke U, Winter G, Moll K, Wheelock CE, Bayarugaba J, Kironde F, Egwang TG, Chen Q, Andersson B, Wahlgren M (2007). PfEMP1-DBL1alpha amino acid motifs in severe disease states of *Plasmodium falciparum *malaria. Proc Natl Acad Sci USA.

[B38] Baruch D, Gamain B, Barnwell J, Sullivan J, Stowers A, Galland G, Miller L, Collins W (2002). Immunization of Aotus monkeys with a functional domain of the *Plasmodium falciparum *variant antigen induces protection against a lethal parasite line. Proc Natl Acad Sci USA.

[B39] Salanti A, Dahlback M, Turner L, Nielsen MA, Barfod L, Magistrado P, Jensen ATR, Lavstsen T, Ofori MF, Marsh K, Hviid L, Theander TG (2004). Evidence for the involvement of VAR2CSA in pregnancy-associated malaria. J Exp Med.

[B40] Viebig NK, Gamain B, Scheidig C, Lepolard C, Przyborski J, Lanzer M, Gysin J, Scherf A (2005). A single member of the *Plasmodium falciparum *var multigene family determines cytoadhesion to the placental receptor chondroitin sulphate A. EMBO Rep.

[B41] Gamain B, Smith J, Viebig N, Gysin J, Scherf A (2007). Pregnancy-associated malaria: parasite binding, natural immunity and vaccine development. Int J Parasitol.

[B42] Recker M, Nee S, Bull P, Kinyanjui S, Marsh K, Newbold C, Gupta S (2004). Transient cross-reactive immune responses can maintain antigenic variation in malaria. Nature.

[B43] Gupta S, Maiden M, Feavers I, Nee S, May R, Anderson R (1996). The maintenance of strain structure in populations of recombining infectious agents. Nat Med.

[B44] Gupta S, Ferguson N, Anderson R (1998). Chaos, persistence and the evolution of strain structure in populations of antigenically variable infectious agents. Science.

[B45] Riley EM, Allen SJ, Wheeler JG, Blackman MJ, Bennett S, Takacs B, Schonfeld HJ, Holder AA, Greenwood BM (1992). Naturally acquired cellular and humoral immune responses to the major merozoite surface antigen (PfMSP1) of *Plasmodium falciparum *are associated with reduced malaria morbidity. Parasite Immunol.

[B46] al Yaman F, Genton B, Kramer K, Chang S, Hui G, Baisor M, Alpers M (1996). Assessment of the role of naturally acquired antibody levels to *Plasmodium falciparum *merozoite surface protein-1 in protecting Papua New Guinean children from malaria morbidity. Am J Trop Med Hyg.

[B47] Roussilhon C, Oeuvray C, Muller-Graf C, Tall A, Rogier C, Trape J, Theisen M, Balde A, Perignon J, Druilhe P (2007). Long-term clinical protection from *falciparum *malaria is strongly associated with IgG3 antibodies to merozoite surface protein 3. PLoS Med.

[B48] Fowler E, Peters J, Gatton M, Chen N, Cheng Q (2002). Genetic diversity of the DBLalpha region in *Plasmodium falciparum *var genes among Asia-Pacific isolates. Mol Biochem Parasitol.

[B49] Tami A, Ord R, Targett G, Sutherland C (2003). Sympatric *Plasmodium falciparum *isolates from Venezuela have structured var gene repertoires. Malar J.

